# A health security-based framework for prioritizing regions for digital learning in complex health emergencies

**DOI:** 10.3389/fpubh.2024.1455470

**Published:** 2025-01-09

**Authors:** Shawn M. D’Andrea, Nada Fadul, Bruce Struminger

**Affiliations:** ^1^Brigham and Women’s Hospital, Boston, MA, United States; ^2^Department of Internal Medicine, University of Nebraska Medical Center College of Medicine, Omaha, NE, United States; ^3^ECHO Institute and Department of Internal Medicine, University of New Mexico Health Sciences Center, Albuquerque, NM, United States

**Keywords:** digital learning, health emergencies, health security, health security index, regional prioritization, humanitarian response

## Abstract

Digital health and learning have expanded significantly in recent decades though their use in settings of acute health emergencies has only recently begun. Growing experience among organizations working in the digital health and learning space suggest that virtual communities of practice in these areas may have value in response to health emergencies. Evaluation of recent virtual programs applied in acute health emergencies suggest that a pre-established digital learning network can serve as a valuable resource when an acute health emergency strikes. This paper introduces the concept, and explores the potential value of developing a prioritization framework, informed by health security assessments, to identify countries most vulnerable to future health crises. By using an anticipatory approach and framework to identify high risk regions, digital learning programs can be developed proactively, building networks that can be activated during emergencies. Creating and prioritizing virtual learning networks in regions at high risk of health emergencies can improve response capacities. Developing a framework to identify countries and regions of greatest risk can help policy makers, educators, and donors, focus limited resources on high need areas.

## Introduction

Digital health has rapidly expanded in the 21st century, with telemedicine, remote clinical encounters, and digital education experiencing significant growth, especially during the COVID-19 pandemic. Public health emergencies encompass a range of population level health events such as epidemics or pandemics, natural disasters, and wide-ranging health effects of war and conflict on public health. Digital learning in humanitarian or public health emergencies is a more recent application of this educational approach. In the past several years organizations such as Project ECHO at the University of New Mexico, a WHO Collaborating Centre for Digital Learning in Health Emergencies, and Health Tech Without Borders (HTWB) which has provided both education and tele-health services in crisis settings, have supported countries facing epidemic and pandemic response (1) as well as humanitarian emergencies including armed conflicts (2, 3) and natural disasters (4).

As adaptation of digital learning in the response to health emergencies grows, a vulnerability assessment-based approach to prioritize countries and regions most likely to benefit from such resources may help optimize the benefit of these programs. By using pre-existing, or *de novo*, assessments of the health security status of countries, states, and regions, those at high risk of future health emergencies, and with critical preparedness gaps, can be identified. This approach can help policymakers, donors, and digital learning program leaders allocate limited resources and attention more effectively when developing digital learning infrastructure and programs to ensure the ability to respond rapidly to health emergencies.

## Recent lessons learned: digital learning in Ukraine and Sudan

While digital learning for health has been an educational practice for decades, it has recently been leveraged to enhance emergency medical care capacity in active conflict settings. In June 2022, Project ECHO, in collaboration with the Swiss Foundation for Innovation and the Ministry of Health of Ukraine, rapidly developed and deployed an eight-week real-time, interactive, virtual trauma care training program based on the World Health Organization/International Committee of the Red Cross (WHO/ICRC) Basic Emergency Care (BEC) Trauma Module to teach trauma care to non-emergency trained healthcare workers (5)(HCWs). Similarly, following the outbreak of hostilities in Sudan in April 2023, a virtual learning program was developed within one week to provide emergency care training to a wide range of healthcare professionals affected by the conflict (3).

These programs offer important lessons for using limited digital learning resources for acute health emergencies in high-risk settings. For instance, the Sudan program contrasted with the Ukraine training as it was deployed on a pre-existing virtual network of healthcare providers organized through an established digital learning program. This Community Medical Response Team (CMRT) program, organized by the Sustainable Development Response Organization (SUDRO), had been established to help respond to the COVID-19 pandemic and was a successful virtual community of practice for several years, offering primary care training and education to medical students and HCWs in Sudan. With the outbreak of war in April 2023, a real-time, interactive digital emergency care training program was rapidly coordinated, providing emergency medical and trauma training to Sudanese healthcare workers within the CMRT network. The time from conception to activation of this program was less than one week. Prior to launch of the emergency care training program the CMRT network included approximately 5,000 Sudanese HCWs. When the training was announced this network expanded to over 14,000 healthcare workers who were then able to receive communication from program organizers including announcements of upcoming digital learning sessions and didactic materials which could be shared through the Telegram Messenger application. The program’s success in reaching many HCWs in an active conflict zone highlights the value of creating pre-established virtual learning networks in high-risk countries. These networks empower response and support organizations to coordinate education and resource sharing, including psychosocial support, with local healthcare workers during emergencies.

## A global strategy for prioritization of digital learning in health emergencies

As the use of digital learning in health emergencies grows, developing a framework for prioritizing countries and regions for investment in digital health education programming is critical for proactive capacity building and enhancing effectiveness of response efforts. By creating these digital learning programs in advance of a crisis, an important virtual infrastructure is developed; this results in a digitally connected network of health professionals ready to be activated and leveraged for teaching and information sharing before and during a health emergency.

## Digital learning: health system assessment

With limited financial resources and attention available to develop such learning networks globally, an approach that prioritizes regions at the greatest risk of a large-scale health crisis is needed. This prioritization should be based on a recent health system assessment. Recognizing that all indices and evaluation frameworks will have some limitations, cross referencing results of multiple assessment models such as the World Health Organization Joint External Evaluation (6) (JEE) score or the Global Health Security Index (7) ranking may be of value. These assessments offer a comparative analysis of baseline health security and provide potential data to guide digital learning in health emergency priorities. Other indices that could be used include the ND GAIN Index (8) for climate vulnerability and the Fragile States Index 2024 (9). A convened workgroup of representatives of key stakeholder groups could review existing indices of health sector capacity, and specific vulnerability assessments (e.g., climate) of nations to first determine whether existing measures of health sector preparedness and capacity to respond to health emergencies sufficiently identify those regions most likely to benefit from a digital health infrastructure. After an initial review a determination should be made to proceed with use of existing health capacity or vulnerability index, or to develop a new assessment tool to rank nations according to likelihood of benefiting from a digital learning for health program.

Limitations to the use of an existing framework include a failure of the index to correctly identify a region likely to benefit from a digital learning program to provide benefit in a health emergency. In contrast, developing a new index could prove cumbersome, time-consuming, and highly specific to the subject of digital learning which could call into question the value of time and resources spent on developing another health system assessment index. These pitfalls highlight the importance of thoughtful stakeholder selection and robust discourse among stakeholders when determining whether to rely on existing health system assessments or creating a health system assessment for digital learning *de novo*.

## Prioritization of regions most likely to benefit from digital learning programs

The development of a more standardized and efficient prioritization scheme could be catalyzed by request to the United Nations/World Health Organization (UN/WHO) to create such a priority list, which would be based on their assessment of indicators to assist with the prioritization of digital learning anticipatory action. Collaboration between leaders of digital learning in healthcare and the UN/WHO in identifying such a priority list might lead to a classification of priority countries and regions more specifically focused on settings whose needs and vulnerabilities may be more responsive to the benefits of digital learning networks. The prioritization scheme should be based on an assessment that helps identify regions at increased risk of health emergencies and with resource limitations where increased investment in a virtual learning network is most likely to be of value. For example, a country or region with relatively high resources and low risk of population wide health emergency such as war or natural disaster might benefit less from development of a virtual learning network as the country would be able to mitigate the impact of the health emergency with existing resources. In contrast a country with fewer resources and greater risk of a health emergency would likely have greater benefit of implementation of a virtual learning network in the event of a health emergency providing an avenue for rapid education and information sharing among healthcare personnel.

In addition, prioritizing the delivery of digital learning and health emergency programming to high-risk countries or regions provides a more objective and need-based strategy for program development. Given limited donor funding for public health programs, using an objective approach, such as a prioritization of countries/regions by a UN/WHO led initiative, to determine areas at greatest risk can help triage digital learning resource allocation at a global level. Importantly the deployment of funds to support the development of such virtual learning networks should, ideally, be in times of stability for a country or region allowing for a methodical and controlled development of digital learning networks and programs, and not withheld to deliver only in times of crisis.

## Benefits of developing virtual learning networks in settings at high risk of future health emergencies

The potential benefits of having pre-established virtual learning networks during health emergencies are many. The timeline of implementation for such programs, outside of a crisis could be as rapid as weeks to months. As observed in the Sudan program, the ability to very rapidly coordinate and deploy digital learning opportunities during a humanitarian emergency was greatly facilitated and accelerated by the presence of a pre-established continuing professional development virtual learning network, program coordinators, and subject matter experts for health worker capacity building. Potential benefits of anticipatory digital learning communities to enhance country readiness include:

1. **Familiarity with the Learning Platform**: Participants already familiar with the learning delivery platform may be more inclined to participate in a program.
2. **Experienced Coordinators**: Having program coordinators already identified and experienced in producing and delivering digital education sessions to an established learning network eliminates the need to train new teams and speeds deployment of programs. Experienced coordinators would ideally have a cultural and contextual familiarity which would aid communication and foster trust among learners.
3. **Efficient Communication and Feedback**: Familiarity of the digital learning program and learning model allow for efficient communication and feedback to program leaders. This allows for rapid adaptation of program content to meet the changing needs of learners. For example, in the Sudan program an increase in home births during the early days of the war due to unsafe hospital access was quickly communicated to program leaders, allowing for the rapid deployment of maternity care content.
4. **Rapid deployment mechanism for pre-existing guidance:** Extensive content and educational materials, such as standardized management of emergency conditions from the WHO, currently exists and its dissemination for just-in-time information sharing is accelerated when digital learning networks are in place.
5. **Improved access to psychological support:** Psychosocial support and tools such as psychological first aid resources can be more quickly brought to first contact healthcare workers with a pre-established digital learning network in place.
6. **Potential crowdsourcing health sector information**: While the focus of information movement in digital learning is typically from subject matter experts to learners, bidirectional information sharing offers a valuable opportunity for healthcare leaders. A standing network of HCW’s dispersed geographically, though connected digitally, may facilitate information gathering from the health sector related to capacity and capability as well as offer just-in-time access to epidemiological data which may inform response to health emergencies.
7. **Agile platform able to expand and contract to meet need of the crisis**: Based as an online community reaching participants through individual devices, this virtual network has the ability to adapt its scope to meet the need of a given crisis. Programs may be limited in focus on individual professional groups or communities or expanded across multiple communities and professional groups.
8. **Decreased Cost**: Once a virtual learning network is established, there is little cost in deploying new information or programs over the existing network. Cost would be limited to the time of coordinators and experts involved and related to the use of cellular or Wi-Fi data.

Another general benefit of developing virtual learning networks irrespective of predetermined level of risk is the potential to rapidly and broadly expand local networks by connecting with diaspora who are culturally and contextually familiar with the setting. These diaspora experts can participate as program developers and content experts, helping to overcome challenges faced by digital educators unfamiliar with the local culture or healthcare context.

## Inequity in access to digital technology

Finally, an important consideration is incorporating strategies to overcome the digital divide (10) in education, especially for HCWs in low and middle-income countries and conflict zones. Understanding the availability and accessibility of the telecommunications infrastructure and resources is an essential step in establishing digital learning programs. Ensuring access to internet and technology equipment for individuals or groups of learners is critical to address inequities in digital learning. Where gaps in technological resources and infrastructure exist programs should be designed to function within existing capabilities (i.e., using cellular data and relying on low bandwidth platforms) and include pathways for expanding and adapting programs when infrastructure growth allows. This ability to adapt programing based on technological resources and limitations will be key to maximizing sustainability of such programs.

## Conclusion

With an increasing role in responding to health emergencies, digital learning has demonstrated success in several recent capacity-strengthening programs targeting learners based in environments experiencing complex humanitarian emergencies. Experience from these programs highlights the value of pre-established, virtual learning networks composed of culturally competent and context-aware experts. These experiences suggest that advance development of virtual learning networks in settings at high risk of future health emergencies can enhance system response capacity.

A global strategy based on health security, or other relevant vulnerability assessments to identify and prioritize settings in greatest need of health emergency virtual learning networks, using a combination of objective tools may provide a viable framework for organizations, donors, and digital learning leaders. Such a framework should be developed with an intent to identify countries or regions most likely to benefit from the value provided by a digital learning infrastructure. Prioritization of regions of greatest need could help providers of education and training to strengthen humanitarian response efforts to shift from a reactive to a proactive, more anticipatory planning model, building capacity for populations at risk of future health emergencies.

## Data Availability

The original contributions presented in the study are included in the article/supplementary material, further inquiries can be directed to the corresponding author/s.

## References

[1] Hunt RC Struminger BB Redd JT Herrmann J Jolly BT Arora S. Virtual peer-to-peer learning to enhance and accelerate the health system response to COVID-19: the HHS ASPR project ECHO COVID-19 clinical rounds initiative. Ann Emerg Med. (2021) 78:223–8. doi: 10.1016/j.annemergmed.2021.03.035, PMID: 34325856 PMC8052469

[2] Lee J Petrea-Imenokhoeva M Naim H Balk A He S. Rapid deployment of telehealth in a conflict zone: supporting the humanitarian needs in Ukraine. NEJM Catalyst Innovations Care Deliver (2023) 4:CAT-22. doi: 10.1056/cat.22.0413

[3] D’Andrea SM Fadul N Dery M Brim WL Israel AM Struminger BB. Healthcare capacity strengthening in conflict settings through virtual emergency medical training and outreach: Ukraine and Sudan case studies. *Frontiers*. Public Health. (2024) 12:12. doi: 10.3389/fpubh.2024.1441322, PMID: 39494069 PMC11528706

[4] HTWOB. Available at: https://www.htwb.org/pakistan-flood-response#:~:text=Since%20June%202022%2C%20unprecedented%20monsoon,of%20them%20children%2Dhave%20died (Accessed June 26, 2024).

[5] World Health Organization. WHO-ICRC Basic emergency care: approach to the acutely ill and injured. (2018). Available at: https://www.who.int/publications/i/item/9789241513081 (Accessed June 26, 2024).

[6] World Health Organization. Joint external evaluation tool: International Health Regulations (2005), third edition. (2022). Available at: https://www.who.int/publications/i/item/9789240051980 (Accessed January 3, 2025).

[7] Global Health Security Index. (2021). Available at: https://ghsindex.org/ (Accessed June 26, 2024).

[8] ND Gain Index. (2024). Available at: https://gain.nd.edu/our-work/country-index/rankings/ (Accessed June 26, 2024).

[9] Fragile States Index 2024. (2024). Available at: https://worldpopulationreview.com/country-rankings/fragile-states-index (Accessed June 26, 2024).

[10] Reddy H Joshi S Joshi A Wagh V. A critical review of global digital divide and the role of technology in healthcare. Cureus. (2022) 14:e29739. doi: 10.7759/cureus.29739, PMID: 36340549 PMC9621721

